# Non-Quadratic Distances in Model Assessment

**DOI:** 10.3390/e20060464

**Published:** 2018-06-14

**Authors:** Marianthi Markatou, Yang Chen

**Affiliations:** Department of Biostatistics, University at Buffalo, Buffalo, NY 14214, USA

**Keywords:** model assessment, statistical distance, non-quadratic distance, total variation, mixture index of fit, Kullback-Leibler distance, divergence measure

## Abstract

One natural way to measure model adequacy is by using statistical distances as loss functions. A related fundamental question is how to construct loss functions that are scientifically and statistically meaningful. In this paper, we investigate non-quadratic distances and their role in assessing the adequacy of a model and/or ability to perform model selection. We first present the definition of a statistical distance and its associated properties. Three popular distances, total variation, the mixture index of fit and the Kullback-Leibler distance, are studied in detail, with the aim of understanding their properties and potential interpretations that can offer insight into their performance as measures of model misspecification. A small simulation study exemplifies the performance of these measures and their application to different scientific fields is briefly discussed.

## 1. Introduction

Model assessment, that is assessing the adequacy of a model and/or ability to perform model selection, is one of the fundamental components of statistical analyses. For example, in the model adequacy problem one usually begins with a fixed model and interest centers on measuring the model misspecification cost. A natural way to create a framework within which we can assess model misspecification is by using statistical distances as loss functions. These constructs measure the distance between the unknown distribution that generated the data and an estimate from the data model. By identifying statistical distances as loss functions, we can begin to understand the role distances play in model fitting and selection, as they become measures of the overall cost of model misspecification. This strategy will allow us to investigate the construction of a loss function as the maximum error in a list of model fit questions. Therefore, our fundamental question is the following. How can one design a loss function ρ that is scientifically and statistically meaningful? We would like to be able to attach a specific scientific meaning to the numerical values of the loss, so that a value of the distance equal to 4, for example, has an explicit interpretation in terms of our statistical goals. When we select between models, we would like to measure the quality of the approximation via the model’s ability to provide answers to important scientific questions. This presupposes that the meaning of “best fitting model” should depend on the “statistical questions” being asked of the model.

Lindsay [1] discusses a distance-based framework for assessing model adequacy. A fundamental tenet of the framework for model adequacy put forward by Lindsay [1] is that it is possible and reasonable to carry out a model-based scientific inquiry without believing that the model is true, and without assuming that the truth is included in the model. All this of course, assuming that we have a way to measure the quality of the approximation to the “truth”, is offered by the model. This point of view never assumes the correctness of the model. Of course, it is rather presumptuous to label any distribution as the truth as any basic modeling assumption generated by the sampling scheme that provided the data is never exactly true. An example of a basic modeling assumption might be “$X_{1},X_{2},\cdots ,X_{n}$ are independent, identically distributed from an unknown distribution τ”. This, as any other statistical assumption, is subject to question even in the most idealized of data collection frameworks. However, we believe that well designed experiments can generate data that is similar to data from idealized models, therefore we operate as if the basic assumption is true. This means that we assume that there is a true distribution τ that generates the data, which is “knowable” if we can collect an infinite amount of data. Furthermore, we note that the basic modeling assumption will be the global framework for assessment of all more restrictive assumptions about the data generation mechanism. In a sense, it is the “nonparametric” extension of the more restrictive models that might be considered.

We let P be the class of all distributions consistent with the basic assumptions. Hence τ∈P, and sets H∈P are called models. We assume that τ∉H; hence, there is a permanent model misspecification error. Statistical distances will then provide a measure for the model misspecification error.

One natural way to measure model adequacy is to define a loss function ρ(τ,M) that describes the loss incurred when the model element *M* is used instead of the true distribution τ. Such a loss function should, in principle, indicate, in an inferential sense, how far apart the two distributions τ, *M* are. In the next section, we offer a formal definition of the concept of a statistical distance.

If the statistical questions of interest can be expressed as a list of functionals T(M) of the model *M* that we wish to be uniformly close to the same functionals T(τ) of the true distribution, then we can turn the set of model fit questions into a distance via

$$\rho \left(\tau ,M\right)=\underset{T(\cdot )}{\sup}\left|T\left(\tau \right)-T\left(M\right)\right|,$$

where the supremum is taken over the class of functionals of interest. Using the supremum of the individual errors is one way of assessing overall error, but using this measure has the nice feature that its value gives a bound on all individual errors. The statistical questions of interest may be global, such as: is the normal model correct in every aspect? Or we may be interested to have answers on a few key characteristics, such as the mean.

Lindsay et al. [2] introduced a class of statistical distances, called quadratic distances, and studied their use in the context of goodness-of-fit testing. Furthermore, Markatou et al. [3] discuss extensively the chi-squared distance, a special case of quadratic distance, and its role in robustness. In this paper, we study non-quadratic distances and their role in model assessment. The paper is organized as follows. Section 2 presents the definition of a statistical distance and its associated properties. Section 3, Section 4 and Section 5 discuss in detail three popular distances, total variation, the mixture index of fit and the Kullback-Leibler distance, with the aim of understanding their role in model assessment problems. The likelihood distance is also briefly discussed in Section 5. Section 6 illustrates computation and applications of total variation, mixture index of fit and Kullback-Leibler distances. Finally, Section 7 presents discussion and conclusions pertaining to the use of total variation and mixture index of fit distances.

## 2. Statistical Distances and Their Properties

If we adopt the usual convention that loss functions are nonnegative in their arguments, and zero if the correct model is used, and have larger value if the two distributions are not very similar, then the loss ρ(τ,M) can also be viewed as a distance between τ, *M*. In fact, we will always assume that for any two distributions *F*, *G*

ρ(F,G)≥0withρ(F,F)=0.

If this holds, we will say that ρ is a statistical distance. Unlike the requirements for a metric, we do not require symmetry. In fact, there is no reason that the loss should be symmetric, as the roles of τ, *M* are different. We also do not require ρ to be nonzero when the arguments differ. This zero property will allow us to specify that two distributions are equivalent as far as our statistical purposes are concerned by giving them zero distance.

Furthermore, it is important to note that if τ is in H and $\tau =M_{\theta_{0}}$, and M∈H, say $M_{\theta }$, then the distance ρ(τ,M) induces a loss function on the parameter space via

$$L_{\rho }\left(\theta_{0},\theta \right)\overset{\mathrm{def}}{=}\rho \left(M_{\theta_{0}},M_{\theta }\right).$$

Therefore, if τ is in the model, the losses defined by ρ are parametric losses.

We begin within the discrete distribution framework. Let T={0,1,2,⋯,T}, where *T* is possibly infinite, be a discrete sample space. On this sample space we define a true probability density τ(t), as well as a family of densities $M=\{m_{\theta }\left(t\right):\theta \in \Theta \}$, where Θ is the parameter space. Assume we have independent and identically distributed random variables $X_{1},X_{2},\cdots ,X_{n}$ producing the realizations $x_{1},x_{2},\cdots ,x_{n}$ from τ(·). We record the data as d(t)=n(t)/n, where n(t) is the number of observations in the sample with value equal to *t*. We note here that we use the word “density” in a generic fashion that incorporates both, probability mass functions as well as probability density functions. A rather formal definition of the concept of statistical distance is as follows.

**Definition** **1.** *(Markatou et al. [3]) Let τ, m be two probability density functions. Then ρ(τ,m) is a statistical distance between the corresponding probability distributions if ρ(τ,m)≥0, with equality if and only if τ and m are the same for all statistical purposes.*

We would require ρ(τ,m) to indicate the worst mistake that we can make if we use *m* instead of τ. The precise meaning of this statement is obvious in the case of total variation that we discuss in detail in Section 3 of the paper.

We would also like our statistical distances to be convex in their arguments.

**Definition** **2.** *Let τ, m be a pair of probability density functions, with m being represented as $m=\alpha m_{1}+\left(1-\alpha \right)m_{2}$, 0≤α≤1. We say that the statistical distance ρ(τ,m) is convex in the right argument if*

$$\rho \left(\tau ,\alpha m_{1}+\left(1-\alpha \right)m_{2}\right)\leq \alpha \rho \left(\tau ,m_{1}\right)+\left(1-\alpha \right)\rho \left(\tau ,m_{2}\right),$$

*where $m_{1}$, $m_{2}$ are two probability density functions.*

**Definition** **3.** *Let τ, m be a pair of probability density functions, and assume $\tau =\gamma \tau_{1}+\left(1-\gamma \right)\tau_{2}$, 0≤γ≤1. Then, we say that ρ(τ,m) is convex in the left argument if*

$$\rho \left(\gamma \tau_{1}+\left(1-\gamma \right)\tau_{2},m\right)\leq \gamma \rho \left(\tau_{1},m\right)+\left(1-\gamma \right)\rho \left(\tau_{2},m\right),$$

*where $\tau_{1}$, $\tau_{2}$ are two densities.*

Lindsay et al. [2] define and study quadratic distances as measures of goodness of fit, a form of model assessment. In the next sections, we study non-quadratic distances and their role in the problem of model assessment. We begin with the total variation distance.

## 3. Total Variation

In this section, we study the properties of the total variation distance. We offer a loss function interpretation of this distance and discuss sensitivity issues associated with its use. We will begin with the case of discrete probability measures and then move to the case of continuous probability measures. The results presented here are novel and are useful in selecting the distances to be used in any given problem.

The total variation distance is defined as follows.

**Definition** **4.** *Let τ, m be two probability distributions. We define the total variation distance between the probability mass functions τ, m to be*

$$V\left(\tau ,m\right)=\frac{1}{2}\sum_{t}\left|\tau (t)-m(t)\right|.$$

This measure is also known as the $L_{1}$-distance (without the factor 1/2) or index of dissimilarity.

**Corollary** **1.** *The total variation distance takes values in the interval [0,1].*

**Proof.**  By definition V(τ,m)≥0 with equality if and only if τ=m, ∀t. Moreover, $\left|\tau (t)-m(t)\right|\leq \left|\tau (t)\right|+\left|m(t)\right|$. But τ, *m* are probability mass functions (or densities), therefore

$$\left|\tau (t)-m(t)\right|\leq \tau \left(t\right)+m\left(t\right)$$

and hence

$$\frac{1}{2}\sum_{t}\left|\tau (t)-m(t)\right|\leq \frac{1}{2}\left(\sum_{t}\tau \left(t\right)+\sum_{t}m\left(t\right)\right)$$

or, equivalently

$$\frac{1}{2}\sum_{t}\left|\tau (t)-m(t)\right|\leq \frac{1}{2}\left(1+1\right)=1.$$

Therefore 0≤V(τ,m)≤1. ☐

**Proposition** **1.** *The total variation distance is a metric.*

**Proof.**  By definition, the total variation distance is non-negative. Moreover, it is symmetric because V(τ,m)=V(m,τ) and it satisfies the triangle inequality since

$$\begin{array}{cc} V(\tau ,m) & =\frac{1}{2}\sum \left|\tau (t)-m(t)\right|\\ & =\frac{1}{2}\sum \left|\tau (t)-g(t)+g(t)-m(t)\right|\\ & \leq \frac{1}{2}\left(\sum \left|\tau (t)-g(t)\right|+\sum \left|g(t)-m(t)\right|\right)\\ & =V(\tau ,g)+V(g,m). \end{array}$$

Thus, it is a metric. ☐

The following proposition states that the total variation distance is convex in both, left and right arguments.

**Proposition** **2.** *Let τ, m be a pair of densities with τ represented as $\tau =\alpha \tau_{1}+\left(1-\alpha \right)\tau_{2}$, 0≤α≤1. Then*

$$V\left(\alpha \tau_{1}+\left(1-\alpha \right)\tau_{2},m\right)\leq \alpha V\left(\tau_{1},m\right)+\left(1-\alpha \right)V\left(\tau_{2},m\right).$$

*Moreover, if m is represented as $m=\gamma m_{1}+\left(1-\gamma \right)m_{2}$, 0≤γ≤1, then*

$$V\left(\tau ,\gamma m_{1}+\left(1-\gamma \right)m_{2}\right)\leq \gamma V\left(\tau ,m_{1}\right)+\left(1-\gamma \right)V\left(\tau ,m_{2}\right).$$

**Proof.**  It is a straightforward application of the definition of the total variation distance. ☐

The total variation measure has major implications for prediction probabilities. A statistically useful interpretation of the total variation distance is that it can be thought of as the worst error we can commit in probability when we use the model *m* instead of the truth τ. The maximum value of this error equals 1 and it occurs when τ, *m* are mutually singular.

Denote by $P_{\tau }$ the probability of a set under the measure τ and by $P_{m}$ the probability of a set under the measure *m*.

**Proposition** **3.** *Let τ, m be two probability mass functions. Then*

$$V\left(\tau ,m\right)=\underset{A\subset B}{\sup}\left|P_{\tau }\left(A\right)-P_{m}\left(A\right)\right|,$$

*where A is a subset of the Borel set B.*

**Proof.**  Define the sets $B_{1}=\left\{t:\tau (t)>m(t)\right\}$, $B_{2}=\left\{t:\tau (t)<m(t)\right\}$, $B_{3}=\left\{t:\tau (t)=m(t)\right\}$. Notice that

$$\begin{array}{c} P_{\tau }\left(B_{1}\right)+P_{\tau }\left(B_{2}\right)+P_{\tau }\left(B_{3}\right)=P_{m}\left(B_{1}\right)+P_{m}\left(B_{2}\right)+P_{m}\left(B_{3}\right)=1. \end{array}$$

Because on the set $B_{3}$ the two probability mass functions are equal $P_{\tau }\left(B_{3}\right)=P_{m}\left(B_{3}\right)$, and hence

$$P_{\tau }\left(B_{1}\right)-P_{m}\left(B_{1}\right)=P_{m}\left(B_{2}\right)-P_{\tau }\left(B_{2}\right).$$

Note that, because of the nature of the sets $B_{1}$ and $B_{2}$, both terms in the last expression are positive. Therefore

$$\begin{array}{c} \begin{array}{cc} V(\tau ,m) & =\frac{1}{2}\sum \left|\tau (t)-m(t)\right|\\ & =\frac{1}{2}\left(\sum_{t\in B_{1}}\left|\tau (t)-m(t)\right|+\sum_{t\in B_{2}}\left|\tau (t)-m(t)\right|+\sum_{t\in B_{3}}\left|\tau (t)-m(t)\right|\right)\\ & =\frac{1}{2}\left\{\left(P_{\tau }\left(B_{1}\right)-P_{m}\left(B_{1}\right)\right)+\left(P_{m}\left(B_{2}\right)-P_{\tau }\left(B_{2}\right)\right)\right\}\\ & =P_{\tau }\left(B_{1}\right)-P_{m}\left(B_{1}\right). \end{array} \end{array}$$

Furthermore

$$\underset{A\subset B}{\sup}\left|P_{\tau }\left(A\right)-P_{m}\left(A\right)\right|=\max\left\{\underset{A\subset B}{\sup}\left(P_{\tau }\left(A\right)-P_{m}\left(A\right)\right),\underset{A\subset B}{\sup}\left(P_{m}\left(A\right)-P_{\tau }\left(A\right)\right)\right\}.$$

But

$$\underset{A\subset B}{\sup}\left(P_{\tau }\left(A\right)-P_{m}\left(A\right)\right)=P_{\tau }\left(B_{1}\right)-P_{m}\left(B_{1}\right)$$

and

$$\underset{A\subset B}{\sup}\left(P_{m}\left(A\right)-P_{\tau }\left(A\right)\right)=P_{m}\left(B_{2}\right)-P_{\tau }\left(B_{2}\right)=P_{\tau }\left(B_{1}\right)-P_{m}\left(B_{1}\right).$$

Therefore

$$\underset{A\subset B}{\sup}\left|P_{\tau }\left(A\right)-P_{m}\left(A\right)\right|=P_{\tau }\left(B_{1}\right)-P_{m}\left(B_{1}\right),$$

and hence

$$V\left(\tau ,m\right)=\underset{A\subset B}{\sup}\left|P_{\tau }\left(A\right)-P_{m}\left(A\right)\right|.$$

☐

**Remark** **1.** *The model misspecification measure V(τ,m) has a “minimax ”expression*

$$V\left(\tau ,M\right)=\underset{m\in M}{\inf}\underset{A}{\sup}\left\{\left|P_{\tau }\left(A\right)-P_{m}\left(A\right)\right|:A\subset B\right\}.$$

*This indicates the sense in which the measure assesses the overall risk of using m instead of τ, then chooses m that minimizes the aforementioned risk.*

We now offer a testing interpretation of the total variation distance. We establish that the total variation distance can be obtained as a solution to a suitably defined optimization problem. It is obtained as that test function which maximizes the difference between the power and level of a suitably defined test problem.

**Definition** **5.** *A randomized test function for testing a statistical hypothesis $H_{0}$ versus the alternative $H_{1}$ is a (measurable) function ϕ defined on $R^{n}$ and taking values in the interval [0,1] with the following interpretation. If x is the observed value of X and ϕ(x)=y, then a coin whose probability of falling heads is y is tossed and $H_{0}$ is rejected when head appears. In the case where y is either 0 or 1, ∀x, the test is called non-randomized.*

**Proposition** **4.** *Let $H_{0}:\tau \left(x\right)=f\left(x\right)$ versus $H_{1}:\tau \left(x\right)=g\left(x\right)$ and ϕ(x) is a test function, f, g are probability mass functions. Then*

$$V\left(f,g\right)=\max_{\phi }\left\{E_{H_{1}}\left(\phi (X)\right)-E_{H_{0}}\left(\phi (X)\right)\right\}.$$

**Proof.**  We have

$$E_{H_{1}}\left(\phi (X)\right)-E_{H_{0}}\left(\phi (X)\right)=\sum \phi \left(x\right)\left(g(x)-f(x)\right).$$

Then

$$\phi \left(x\right)=1\;\mathrm{if}\;x\in B_{1}=\left\{x:g(x)>f(x)\right\},$$

So

$$\max_{\phi }\sum \phi \left(x\right)\left(g(x)-f(x)\right)=P_{g}\left(B_{1}\right)-P_{f}\left(B_{1}\right)=V\left(f,g\right).$$

☐

An advantage of the total variation distance is that it is not sensitive to small changes in the density. That is, if τ(t) is replaced by τ(t)+e(t) where $\sum_{t}e\left(t\right)=0$ and $\sum_{t}\left|e(t)\right|$ is small then

$$\begin{array}{cc} V(\tau +e,m) & =\frac{1}{2}\sum \left|\tau (t)+e(t)-m(t)\right|\\ & \leq \frac{1}{2}\sum \left|\tau (t)-m(t)\right|+\frac{1}{2}\sum \left|e(t)\right|\\ & =V\left(\tau ,m\right)+\frac{1}{2}\sum \left|e(t)\right|. \end{array}$$

Therefore, when the changes in the density are small V(τ+e,m)≈V(τ,m). When describing a population, it is natural to describe it via the proportion of individuals in various subgroups. Having V(τ,m) small would ensure uniform accuracy for all such descriptions. On the other hand, populations are also described in terms of a variety of other variables, such as means. Having the total variation measure small does not imply that means are close on the scale of standard deviation.

**Remark** **2.** *The total variation distance is not differentiable in the arguments. Using $V(d,m_{\theta })$ as an inference function, where d denotes the data estimate of τ (i.e., $\hat{\tau }$), yields estimators of θ that have the feature of not generating smooth, asymptotically normal estimators when the model is true [4]. This feature is related to the pathologies of the variation distance described by Donoho and Liu [5]. However, if parameter estimation is of interest, one can use alternative divergences that are free of these pathologies.*

We now study the total variation distance in continuous probability models.

**Definition** **6.** *The total variation distance between two probability density functions τ, m is defined as*

$$V\left(\tau ,m\right)=\frac{1}{2}\int \left|\tau (x)-m(x)\right|dx.$$

The total variation distance has the same interpretation as in the discrete probability model case. That is

$$V\left(\tau ,m\right)=\underset{A\subset B}{\sup}\left|P_{\tau }\left(A\right)-P_{m}\left(A\right)\right|.$$

One of the important issues in the construction of distances in continuous spaces is the issue of invariance, because the behavior of distance measures under transformations of the data is of interest. Suppose we take a monotone transformation of the observed variable *X* and use the corresponding model distribution; how does this transformation affect the distance between *X* and the model?

Invariance seems to be desirable from an inferential point of view, but difficult to achieve without forcing one of the distributions to be continuous and appealing to the probability integral transform for a common scale. In multivariate continuous spaces, the problem of transformation invariance is even more difficult, as there is no longer a natural probability integral transformation to bring data and model on a common scale.

**Proposition** **5.** *Let $V(\tau_{X},m_{X})$ be the total variation distance between the densities $\tau_{X}$, $m_{X}$ for a random variable X. If Y=a(X) is a one-to-one transformation of the random variable X, then*

$$V\left(\tau_{X},m_{X}\right)=V\left(\tau_{Y},m_{Y}\right).$$

**Proof.**  Write

$$\begin{array}{cc} V(\tau_{Y},m_{Y}) & =\frac{1}{2}\int \left|\tau_{Y}\left(y\right)-m_{Y}\left(y\right)\right|dy\\ & =\frac{1}{2}\int \left|\tau_{X}\left(b(y)\right)\cdot |\frac{d}{dy}b\left(y\right)|-m_{X}\left(b(y)\right)\cdot \left|\frac{d}{dy}b\left(y\right)\right|\right|dy\\ & =\frac{1}{2}\int \left|\tau_{X}\left(b(y)\right)-m_{X}\left(b(y)\right)\right|\cdot \left|\frac{d}{dy}b\left(y\right)\right|dy, \end{array}$$

where b(y) is the inverse transformation. Next, we do a change of variable in the integral. Set x=b(y) from where we obtain y=a(x) and $dy=a^{\prime }\left(x\right)dx$; the prime denotes derivative with respect to the corresponding argument. Then

$$V\left(\tau_{Y},m_{Y}\right)=\frac{1}{2}\int \left|\tau_{X}\left(x\right)-m_{X}\left(x\right)\right|\cdot \left|b^{\prime }\left(a(x)\right)\right|\cdot a^{\prime }\left(x\right)dx.$$

But

$$\begin{array}{c} b\left(a(x)\right)=x⟹\frac{d}{dx}b\left(a(x)\right)=1\\ ⟹b^{\prime }\left(a(x)\right)a^{\prime }\left(x\right)=1\\ ⟹b^{\prime }\left(a(x)\right)=\frac{1}{a^{\prime }\left(x\right)}, \end{array}$$

hence

$$\begin{array}{cc} V(\tau_{Y},m_{Y}) & =\frac{1}{2}\int \left|\tau_{X}\left(x\right)-m_{X}\left(x\right)\right|\cdot \frac{a^{\prime }\left(x\right)}{|a^{\prime }\left(x\right)|}dx\\ & =\frac{1}{2}\int \left|\tau_{X}\left(x\right)-m_{X}\left(x\right)\right|\cdot sign\left(a^{\prime }\left(x\right)\right)dx. \end{array}$$

Now since a(·) is a one-to-one transformation, a(x) is either increasing or decreasing on different segments of R. Thus

$$V\left(\tau_{Y},m_{Y}\right)=V\left(\tau_{X},m_{X}\right),$$

where Y=a(X). ☐

A fundamental problem with the total variation distance is that it cannot be used to compute the distance between a discrete distribution and a continuous distribution because the total variation distance between a continuous measure and a discrete measure is always the maximum possible, that is 1. This inability of the total variation distance to discriminate between discrete and continuous measures can be interpreted as asking “too many questions”at once, without any prioritization. This limits its use despite its invariant characteristics.

We now discuss the relationship between the total variation distance and Fisher information. Denote by $m^{\left(n\right)}$ the joint density of *n* independent and identically distributed random variables. Then we have the following proposition.

**Proposition** **6.** *The total variation distance is locally equivalent to the Fisher information number, that is*

$$\frac{1}{n}V\left(m_{\theta }^{\left(n\right)},m_{\theta_{0}}^{\left(n\right)}\right)\to \left|\theta -\theta_{0}\right|\sqrt{\frac{I(\theta_{0})}{2\pi }},\;\mathrm{as}\;n\to \infty ,$$

*where $m_{\theta }$, $m_{\theta_{0}}$ are two discrete probability models.*

**Proof.**  By definition

$$V\left(m_{\theta }^{\left(n\right)},m_{\theta_{0}}^{\left(n\right)}\right)=\frac{1}{2}\sum \left|m_{\theta }^{\left(n\right)}\left(t\right)-m_{\theta_{0}}^{\left(n\right)}\left(t\right)\right|.$$

Now, expand $m_{\theta }^{\left(n\right)}\left(t\right)$ using Taylor series in the neighborhood of $\theta_{0}$ to obtain

$$m_{\theta }^{\left(n\right)}\left(t\right)≃m_{\theta_{0}}^{\left(n\right)}\left(t\right)+\left(\theta -\theta_{0}\right){\left(m_{\theta_{0}}^{\left(n\right)}\left(t\right)\right)}^{\prime },$$

where the prime denotes derivative with respect to the parameter θ. Further, write

$${\left(m_{\theta_{0}}^{\left(n\right)}\left(t\right)\right)}^{\prime }=m_{\theta_{0}}^{\left(n\right)}\left(t\right)\left(\frac{d}{d\theta }\log m_{\theta }^{\left(n\right)}\left(t\right){|}_{\theta_{0}}\right)$$

to obtain

$$\begin{array}{cc} \frac{1}{n}V\left(m_{\theta }^{\left(n\right)},m_{\theta_{0}}^{\left(n\right)}\right) & ≃\frac{1}{2}\left|\theta -\theta_{0}\right|E\left\{\frac{1}{n}|\frac{d}{d\theta }\log m_{\theta }^{\left(n\right)}\left(t\right){|}_{\theta_{0}}|\right\}\\ & =\frac{1}{2}\left|\theta -\theta_{0}\right|E\left\{|\frac{1}{n}\sum_{i=1}^{n}u_{\theta_{0}}\left(t_{i}\right)|\right\}, \end{array}$$

where

$$u_{\theta_{0}}\left(t_{i}\right)=\frac{d}{d\theta }\log m_{\theta }\left(t_{i}\right){|}_{\theta_{0}}.$$

Therefore, assuming that $\frac{1}{n}\sum u_{\theta_{0}}\left(t_{i}\right)$ converges to a normal random variable in absolute mean, then

$$\frac{1}{n}V\left(m_{\theta }^{\left(n\right)},m_{\theta_{0}}^{\left(n\right)}\right)\to \frac{1}{2}|\theta -\theta_{0}|\sqrt{I(\theta_{0})}\sqrt{\frac{2}{\pi }}=\left|\theta -\theta_{0}\right|\sqrt{\frac{I(\theta_{0})}{2\pi }},\;\mathrm{as}\;n\to \infty ,$$

because $E\left(u_{\theta_{0}}\left(t_{i}\right)\right)=0$, $\mathrm{Var}\left(u_{\theta_{0}}\left(t_{i}\right)\right)=I\left(\theta_{0}\right)$ and $E\left(|Z|\right)=\sqrt{\frac{2}{\pi }}$ when Z∼N(0,1). ☐

The total variation is a non-quadratic distance. It is however related to a quadratic distance, the Hellinger distance, defined as $H^{2}\left(\tau ,m\right)=\frac{1}{2}\sum {\left(\sqrt{\tau (t)}-\sqrt{m(t)}\right)}^{2}$ by the following inequality.

**Proposition** **7.** *Let τ, m be two probability mass functions. Then*

$$0\leq H^{2}\left(\tau ,m\right)\leq V\left(\tau ,m\right)\leq {\left[H^{2}\left(\tau ,m\right)\left(2-H^{2}\left(\tau ,m\right)\right)\right]}^{\frac{1}{2}}.$$

**Proof.**  Straightforward using the definitions of the distances involved and Cauchy-Swartz inequality. Holder’s inequality provides $1-\sum \sqrt{\tau (t)m(t)}\geq 0$. ☐

Note that $2H^{2}\left(\tau ,m\right)=\sum {\left[\sqrt{\tau (t)}-\sqrt{m(t)}\right]}^{2}$; the square root of this quantity, that is ${\left\{\sum {\left[\sqrt{\tau (t)}-\sqrt{m(t)}\right]}^{2}\right\}}^{1/2}$, is known as Matusita’s distance [6,7]. Further, define the affinity between two probability densities by

$$\rho \left(\tau ,m\right)=\sum_{t}\tau^{1/2}\left(t\right)m^{1/2}\left(t\right).$$

Then, it is easy to prove that

$$\sum_{t}{\left[\sqrt{\tau (t)}-\sqrt{m(t)}\right]}^{2}=2\left(1-\rho (\tau ,m)\right)\leq V\left(\tau ,m\right)\leq 2{\left\{\sum_{t}{\left[\sqrt{\tau (t)}-\sqrt{m(t)}\right]}^{2}\right\}}^{1/2}.$$

The above inequality indicates the relationship between total variation and Matusita’s distance.

## 4. Mixture Index of Fit

Rudas, Clogg, and Lindsay [8] proposed a new index of fit approach to evaluate the goodness of fit analysis of contingency tables based on the mixture model framework. The approach focuses attention on the discrepancy between the model and the data, and allows comparisons across studies. Suppose M is the baseline model. The family of models which are proposed for evaluating goodness of fit is a two-point mixture model given by

$$M_{\pi }=\left\{\tau :\tau \left(t\right)=\left(1-\pi \right)m_{\theta }\left(t\right)+\pi e\left(t\right),m_{\theta }\left(t\right)\in M,e\left(t\right)\;\mathrm{arbitrary},\theta \in \Theta \right\}.$$

Here π denotes the mixing proportion, which is interpreted as the proportion of the population outside the model M. In the robustness literature the mixing proportion corresponds to the contamination proportion, as explained below. In the contingency table framework $m_{\theta }\left(t\right)$, e(t) describe the tables of probabilities for each latent class. The family of models $M_{\pi }$ defines a class of nested models as π varies from zero to one. Thus, if the model M does not fit well the data, then by increasing π, the model $M_{\pi }$ will be an adequate fit for π sufficiently large.

We can motivate the index of fit by thinking of the population as being composed of two classes with proportions 1−π and π respectively. The first class is perfectly described by M, whereas the second class contains the “outliers”. The index of fit can then be interpreted as the fraction of the population intrinsically outside M, that is, the proportion of outliers in the sample.

We note here that these ideas can be extended beyond the contingency table framework. In our setting, the probability distribution describing the true data generating mechanism may be written as $\tau \left(t\right)=\left(1-\pi \right)m_{\theta }\left(t\right)+\pi e\left(t\right)$, where $m_{\theta }\left(t\right)\in M$ and e(t) is arbitrary. This representation of τ(t) is arbitrary such that we can construct another representation $\tau \left(t\right)=\left(1-\pi -\delta \right)m_{\theta }\left(t\right)+\left(\pi +\delta \right)e^{*}\left(t\right)$. However, there always exists the smallest unique π such that there exists a representation of τ(t) that puts the maximum proportion in one of the population classes. Next, we define formally the mixture index of fit.

**Definition** **7.** (Rudas, Clogg, and Lindsay [8]) The mixture index of fit π* is defined by

$$\pi^{*}\left(\tau \right)=\inf\left\{\pi :\tau \left(t\right)=\left(1-\pi \right)m_{\theta }\left(t\right)+\pi e\left(t\right),m_{\theta }\left(t\right)\in M,e\left(t\right)\;\mathrm{arbitrary}\right\}.$$

Notice that $\pi^{*}\left(\tau \right)$ is a distance. This is because if we set $\pi^{*}\left(\tau ,m_{\theta }\right)=\inf\left\{\pi :\tau \left(t\right)=\left(1-\pi \right)m_{\theta }\left(t\right)+\pi e\left(t\right),e\left(t\right)\;\mathrm{arbitrary}\right\}$ for a fixed $m_{\theta }\left(t\right)$, we have $\pi^{*}\left(\tau ,m_{\theta }\right)$ >0 and $\pi^{*}\left(\tau ,m_{\theta }\right)=0$ if $\tau =m_{\theta }$.

**Definition** **8.** Define the statistical distance $\pi^{*}\left(\tau ,M\right)$ as follows:

$$\pi^{*}\left(\tau ,M\right)=\underset{m\in M}{\inf}\pi^{*}\left(\tau ,m\right).$$

**Remark** **3.** *Note that, to be able to present Proposition 8 below, we have turned arbitrary discrete distributions into vectors. As an example, if the sample space T={0,1,2} and P(X=0)=P(X=1)=P(X=2)=1/3, we write this discrete distribution as the vector ${\left(1/3,1/3,1/3\right)}^{T}$. If, furthermore, we consider the vectors ${\vec{\delta }}_{0}={\left(1,0,0\right)}^{T}$, ${\vec{\delta }}_{1}={\left(0,1,0\right)}^{T}$, and ${\vec{\delta }}_{2}={\left(0,0,1\right)}^{T}$ as degenerate distributions assigning mass 1 at positions 0, 1, 2 then ${\left(1/3,1/3,1/3\right)}^{T}=\frac{1}{3}{\vec{\delta }}_{0}+\frac{1}{3}{\vec{\delta }}_{1}+\frac{1}{3}{\vec{\delta }}_{2}$. This representation of distributions is used in the proof of Proposition 8.*

**Proposition** **8.** The set of vectors $\vec{\tau }$ satisfying the relationship $\pi^{*}\left(\vec{\tau },\vec{m}\right)\leq \pi_{0}$ is a simplex with extremal points $\left(1-\pi_{0}\right)\vec{m}+\pi_{0}{\vec{\delta }}_{i}$, where ${\vec{\delta }}_{i}$ is the vector with 1 at the (i+1)th position and 0 everywhere else.

**Proof.**  Given $\vec{\tau }$ with $\pi^{*}\leq \pi_{0}$, there exists a representation of

$$\vec{\tau }=\left(1-\pi_{0}\right)\vec{m}+\pi_{0}\vec{e}.$$

Write any arbitrary discrete distribution $\vec{e}$ as follows:

$$\vec{e}=e_{0}{\vec{\delta }}_{0}+\cdots +e_{T}{\vec{\delta }}_{T},$$

where $\sum_{i=0}^{T}e_{i}=1$ and $\delta_{i}$ takes the value 1 at the (i+1)th position and the value 0 everywhere else. Then

$$\left(1-\pi_{0}\right)\vec{m}+\pi_{0}\vec{e}=e_{0}\left[\left(1-\pi_{0}\right)\vec{m}+\pi_{0}{\vec{\delta }}_{0}\right]+\cdots +e_{T}\left[\left(1-\pi_{0}\right)\vec{m}+\pi_{0}{\vec{\delta }}_{T}\right],$$

which belongs to a simplex. ☐

**Proposition** **9.** *We have*

$$\pi^{*}\left(\tau ,m\right)=\underset{t}{\sup}\left\{1-\frac{\tau (t)}{m(t)}\right\}=1-\underset{t}{\inf}\left\{\frac{\tau (t)}{m(t)}\right\}.$$

**Proof.**  Define

$$\lambda =1-\underset{t}{\inf}\left\{\frac{\tau (t)}{m(t)}\right\}\;\mathrm{and}\;\bar{\lambda }=1-\lambda .$$

Then

$$\begin{array}{cc} \tau (t)-(1-\lambda )m(t) & =\tau \left(t\right)-\underset{t}{\inf}\left\{\frac{\tau (t)}{m(t)}\right\}m\left(t\right)\\ & =m\left(t\right)\left[\frac{\tau (t)}{m(t)}-\underset{t}{\inf}\left\{\frac{\tau (t)}{m(t)}\right\}\right]\geq 0, \end{array}$$

with equality at some *t*. Let now the error term be

$$e^{*}\left(t\right)=\frac{1}{\lambda }\left[\tau \left(t\right)-\bar{\lambda }m\left(t\right)\right].$$

Then $\tau \left(t\right)=\left(1-\lambda \right)m\left(t\right)+\lambda e^{*}\left(t\right)$ and λ cannot be made smaller without making $e^{*}\left(t\right)$ negative at a point $t_{0}$. This concludes the proof. ☐

**Corollary** **2.** *We have*

$$\pi^{*}\left(\tau ,m\right)=1$$

*if there exists $t_{0}$ such that $\tau (t_{0})=0$ and $m(t_{0})>0$.*

**Proof.**  By Proposition 9 $\pi^{*}\leq 1$, but it equals 1 at $t_{0}$. ☐

One of the advantages of the mixture index of fit is that it has an intuitive interpretation that does not depend upon the specific nature of the model being assessed. Liu and Lindsay [9] extended the results of Rudas et al. [8] to the Kullback-Leibler distance. Computational aspects of the mixture index of fit are discussed in Xi and Lindsay [4] as well as in Dayton [10] and Ispány and Verdes [11].

Finally, a new interpretation to the mixture index of fit was presented by Ispány and Verdes [11]. Let P be the set of probability measures and H⊂P. If *d* is a distance measure on P and $N\left(H,\pi \right)=\left\{Q:Q=(1-\pi )M+\pi R,M\in H,R\in P\right\}$, then $\pi^{*}=\pi^{*}\left(P,H\right)$ is the least non-negative solution of the equation $d\left(P,N\left(H,\pi \right)\right):=\min_{Q\in N(H,\pi )}d\left(P,Q\right)=0$ in π.

Next, we offer some interpretations associated with the mixture index of fit. The statistical interpretations made with this measure are attractive, as any statement based on the model applies to at least $1-\pi^{*}$ of the population involved. However, while the “outlier”model seems interpretable and attractive, the distance itself is not very robust.

In other words, small changes in the probability mass function do not necessarily mean small changes in distance. This is because if $m(t_{0})=\varepsilon$, then a change of ε in $\tau (t_{0})$ from ε to 0 causes $\pi^{*}\left(\tau ,m\right)$ to go to 1. Moreover, assume that our framework is that of continuous probability measures, and that our model is a normal density. If τ(t) is a lighter tailed distribution than our normal model m(t), then

$$\lim_{t\to \infty }\left\{1-\frac{\tau (t)}{m(t)}\right\}=1,$$

and therefore

$$\pi^{*}\left(\tau ,m\right)=\underset{t}{\sup}\left\{1-\frac{\tau (t)}{m(t)}\right\}=1.$$

That is, *light tailed densities* are interpreted as 100% outliers. Therefore, the mixture index of fit measures error from the model in a “one-sided” way. This is in contrast to total variation, which measures the size of “holes” as well as the “outliers” by allowing the distributional errors to be neutral.

In what follows, we show that if we can find a mixture representation for the true distribution then this implies a small total variation distance between the true probability mass function and the assumed model *m*. Specifically, we have the following.

**Proposition** **10.** Let π* be the mixture index of fit. If τ(t)=(1−π)m(t)+πe(t), then

$$V\left(\tau ,m\right)\leq \pi^{*}.$$

**Proof.**  Write

$$\begin{array}{cc} V(\tau ,m) & =\frac{1}{2}\sum \left|\left(1-\pi \right)m\left(t\right)+\pi e\left(t\right)-m\left(t\right)\right|\\ & =\frac{1}{2}\sum \left|\pi \left(e(t)-m(t)\right)\right|\\ & =\frac{1}{2}\sum \pi^{*}\left|e\left(t\right)-m\left(t\right)\right|, \end{array}$$

with $\pi =\pi^{*}$. This is because there always exists the smallest unique π such that τ(t) can be represented as a mixture model.

Thus, the above relationship can be written as

$$\begin{array}{c} V\left(\tau ,m\right)=\frac{1}{2}\pi^{*}\sum \left|e\left(t\right)-m\left(t\right)\right|=\pi^{*}V\left(e,m\right)\leq \pi^{*}. \end{array}$$

☐

There is a mixture representation that connects total variation with the mixture index of fit. This is presented below.

**Proposition** **11.** *Denote by*

$$W\left(\tau ,m\right)=\underset{\pi }{\inf}\left\{\pi :\left(1-\pi \right)\tau \left(t\right)+\pi e_{1}\left(t\right)=\left(1-\pi \right)m\left(t\right)+\pi e_{2}\left(t\right)\right\}.$$

*Then*

$$W\left(\tau ,m\right)=\frac{V(\tau ,m)}{1+V(\tau ,m)}.$$

**Proof.**  Fix τ; for any given *m* let $\left(e_{1},e_{2},\tilde{\pi }\right)$ be a solution to the equation

(1) $$\tilde{\pi }\tau +\left(1-\tilde{\pi }\right)e_{1i}=\tilde{\pi }e_{i}+\left(1-\tilde{\pi }\right)e_{2i},i=1,2,\cdots ,T.$$

Let $q_{1i}=\left(1-\tilde{\pi }\right)e_{1i}$ and $q_{2i}=\left(1-\tilde{\pi }\right)e_{2i}$ and note that since

$$\sum e_{1i}=\sum e_{2i}=1$$

then

$$\sum q_{1i}=\sum q_{2i}=1-\tilde{\pi }.$$

Rewrite now Equation (1) as follows:

$$\begin{array}{c} \tilde{\pi }\tau_{i}+q_{1i}=\tilde{\pi }m_{i}+q_{2i}\\ \Rightarrow q_{2i}-q_{1i}=\tilde{\pi }\left(\tau_{i}-m_{i}\right)\\ \Rightarrow q_{2i}-q_{1i}=\tilde{\pi }{\left(\tau_{i}-m_{i}\right)}^{+}-\tilde{\pi }{\left(\tau_{i}-m_{i}\right)}^{-}, \end{array}$$

where ${\left(x\right)}^{+}=\max\left(x,0\right)$ and ${\left(x\right)}^{-}=-\min\left(x,0\right)$. Thus, ignoring the constraints, every pair $\left(e_{1i},e_{2i}\right)$ satisfying the equation above also satisfies

$$\begin{array}{cc} q_{1i} & =\tilde{\pi }{\left(\tau_{i}-m_{i}\right)}^{-}+\varepsilon_{i},\\ q_{2i} & =\tilde{\pi }{\left(\tau_{i}-m_{i}\right)}^{+}+\varepsilon_{i}, \end{array}$$

for some number $\varepsilon_{i}$. Moreover, such pair must have $\varepsilon_{i}\geq 0$ in order the constraints $q_{1i}\geq 0$, $q_{2i}\geq 0$ to be satisfied. Hence, varying $\varepsilon_{i}$ over $\varepsilon_{i}\geq 0$ gives a class of solutions. To determine $\tilde{\pi }$,

$$\begin{array}{cc} \sum_{i}q_{1i} & =\sum_{i}\left(\tilde{\pi }{\left(\tau_{i}-m_{i}\right)}^{-}+\varepsilon_{i}\right)=1-\tilde{\pi },\\ \sum_{i}q_{2i} & =\sum_{i}\left(\tilde{\pi }{\left(\tau_{i}-m_{i}\right)}^{+}+\varepsilon_{i}\right)=1-\tilde{\pi }, \end{array}$$

and adding these we obtain

$$\begin{array}{c} 2\left(1-\tilde{\pi }\right)=\tilde{\pi }\sum \left|\tau_{i}-m_{i}\right|+2\sum \varepsilon_{i}\\ \Rightarrow 2=\tilde{\pi }\left(2+\sum |\tau_{i}-m_{i}|\right)+2\sum \varepsilon_{i}\\ \Rightarrow 2-2\sum \varepsilon_{i}=\tilde{\pi }\left(2+\sum |\tau_{i}-m_{i}|\right)\\ \Rightarrow \tilde{\pi }=\frac{2-2\sum \varepsilon_{i}}{2+\sum |\tau_{i}-m_{i}|}, \end{array}$$

and the maximum value is obtained when $\sum \varepsilon_{i}=0\Rightarrow \varepsilon_{i}=0,\forall i$. Therefore

$$\begin{array}{c} \tilde{\pi }=\frac{2}{2+\sum |\tau_{i}-m_{i}|}=\frac{1}{1+\frac{1}{2}\sum \left|\tau_{i}-m_{i}\right|}=\frac{1}{1+V(\tau ,m)} \end{array}$$

and so

$$W\left(\tau ,m\right)=\frac{V(\tau ,m)}{1+V(\tau ,m)}.$$

☐

Therefore, for small V(τ,m) the mixture index of fit and the total variation distance are nearly equal.

## 5. Kullback-Leibler Distance

The Kullback-Leibler distance [12] is extensively used in statistics and in particular in model selection. The celebrated AIC model selection criterion [13] is based on this distance. In this section, we present the Kullback-Leibler distance and some of its properties with particular emphasis on interpretations.

**Definition** **9.** *The Kullback-Leibler distance between two densities τ, m is defined as*

$$K^{2}\left(\tau ,m\right)=\sum m\left(t\right)\log\left(\frac{m(t)}{\tau (t)}\right),$$

*or*

$$K^{2}\left(\tau ,m\right)=\int m\left(t\right)\log\left(\frac{m(t)}{\tau (t)}\right)dt.$$

**Proposition** **12.** *The Kullback-Leibler distance is nonnegative, that is*

$$K^{2}\left(\tau ,m\right)\geq 0$$

*with equality if and only if τ(t)=m(t).*

**Proof.**  Write

$$\begin{array}{c} K^{2}\left(\tau ,m\right)=\sum m\left(t\right)\left[\log\left(\frac{m(t)}{\tau (t)}\right)+\frac{\tau (t)}{m(t)}-1\right]=\sum m\left(t\right)\left[-\log\left(\frac{\tau (t)}{m(t)}\right)+\frac{\tau (t)}{m(t)}-1\right]. \end{array}$$

Set $X=\frac{\tau (t)}{m(t)}\geq 0$, then −logX+X−1 is a convex, non-negative function that equals 0 at X=1. Therefore $K^{2}\left(\tau ,m\right)\geq 0$. ☐

**Definition** **10.** *We define the likelihood distance between two densities τ, m as*

$$\lambda^{2}\left(\tau ,m\right)=\sum \tau \left(t\right)\log\left(\frac{\tau (t)}{m(t)}\right).$$

The intuition behind the above expression of the likelihood distance comes from the fact that the log-likelihood in the case of discrete random variables taking $n_{j}$ discrete values, $\sum_{j=1}^{m}n_{j}=n$, *m* is the number of groups, can be written, after appropriate algebraic manipulations, in the above form.

Alternatively, we can write the likelihood distance as

$$\lambda^{2}\left(\tau ,m\right)=\sum m\left(t\right)\left[\frac{\tau (t)}{m(t)}\log\left(\frac{\tau (t)}{m(t)}\right)-\frac{\tau (t)}{m(t)}+1\right],$$

and use this relationship to obtain insight into connections of the likelihood distance with the chi-squared measures studied by Markatou et al. [3].

Specifically, if we write the Pearson’s chi-squared statistic as

$$P^{2}\left(\tau ,m\right)=\sum m\left(t\right){\left[\frac{\tau (t)}{m(t)}-1\right]}^{2},$$

then from the functional relationship $r\log r-r+1\leq {\left(r-1\right)}^{2}$ we obtain that $\lambda^{2}\left(\tau ,m\right)\leq P^{2}\left(\tau ,m\right)$. However, it is also clear from the right tails of the functions that there is no way to bound $\lambda^{2}\left(\tau ,m\right)$ below by a multiple of $P^{2}\left(\tau ,m\right)$. Hence, these measures are not equivalent in the same way that Hellinger distance and symmetric chi-squared are (see Lemma 4, Markatou et al. [3]). In particular, knowing that $\lambda^{2}\left(\tau ,m\right)$ is small is no guarantee that all Pearson *z*-statistics are uniformly small.

On the other hand, one can show by the same mechanism that $S^{2}\leq 2k\lambda^{2}$, where k<32/9 and $S^{2}$ is the symmetric chi-squared distance given as

$$S^{2}\left(\tau ,m\right)=\sum \frac{{\left(\tau (t)-m(t)\right)}^{2}}{\frac{1}{2}\tau \left(t\right)+\frac{1}{2}m\left(t\right)}.$$

It is therefore true that small likelihood distance $\lambda^{2}$ implies small *z*-statistics with blended variance estimators. However, the reverse is not true because the right tail in *r* for $S^{2}$ is of magnitude *r*, as opposed to rlogr for the likelihood distance.

These comparisons provide some feeling for the statistical interpretation of the likelihood distance. Its meaning as a measure of model misspecification is unclear. Furthermore, our impression is that likelihood, like Pearson’s chi-squared is too sensitive to outliers and gross errors in the data. Despite Kullback-Leibler’s theoretical and computational advantages, a point of inconvenience in the context of model selection is the lack of symmetry. One can show that reversing the roles of the arguments in the Kullback-Leibler divergence can yield substantially different results. The sum of the Kullback-Leibler distance and the likelihood distance produces the symmetric Kullback-Leibler distance or J divergence. This measure is symmetric in the arguments, and when used as a model selection measure it is expected to be more sensitive than each of the individual components.

## 6. Computation and Applications of Total Variation, Mixture Index of Fit and Kullback- Leibler Distances

The distances discussed in this paper are used in a number of important applications. Euán et al. [14] use the total variation to detect changes in wave spectra, while Alvarez- Esteban et al. [15] cluster time series data on the basis of the total variation distance. The mixture index of fit has found a number of applications in the area of social sciences. Rudas et al. [8] provided examples of the application of π* to two-way contingency tables. Applications involving differential item functioning and latent class analysis were presented in Rudas and Zwick [16] and Dayton [17] respectively. Formann [18] applied it in regression models involving continuous variables. Finally, Revuelta [19] applied the π* goodness-of-fit statistic to finite mixture item response models that were developed mainly in connection with Rasch models [20,21].

The Kullback-Leibler (KL) distance [12] is fundamental in information theory and its applications. In statistics, the celebrated Akaike information Criterion (AIC) [13,22], widely used in model selection, is based on the Kullback-Leibler distance. There are numerous additional applications of the KL distance in fields such as fluid mechanics, neuroscience, machine learning. In economics, Smith, Naik, and Tsai [23] use KL distance to simultaneously select the number of states and variables associated with Markov-switching regression models that are used in marketing and other business applications. KL distance is also used in diagnostic testing for ruling in or ruling out disease [24,25], as well as in a variety of other fields [26].

Table 1 presents the software, written in R, that can be used to compute the aforementioned distances. Additionally, Zhang and Dayton [27] present a SAS program to compute the two-point mixture index of fit for the two-class latent class analysis models with dichotomous variables. There are a number of different algorithms that can be used to compute the mixture index of fit for contingency tables. Rudas et al. [8] propose to use a standard EM algorithm, Xi and Lindsay [4] use sequential quadratic programming and discuss technical details and numerical issues related to applying nonlinear programming techniques to estimate π*. Dayton [10] discusses explicitly the practical advantages associated with the use of nonlinear programming as well as the limitations, while Pan and Dayton [28] study a variety of additional issues associated with computing π*. Additional algorithms associated with the computation of π* can be found in Verdes [29] and Ispány and Verdes [11].

We now describe a simulation study that aims to illustrate the performance of the total variation, Kullback-Leibler, and mixture index of fit as model selection measures. Data are generated from either an asymmetric $\left(1-\varepsilon \right)N\left(0,1\right)+\varepsilon N\left(\mu ,\sigma^{2}\right)$ contamination model, or from a symmetric $\left(1-\varepsilon \right)N\left(0,1\right)+\varepsilon N\left(0,\sigma^{2}\right)$ contamination model, where ε is the percentage of contamination. Specifically, we generate 500 Monte Carlo samples of sample sizes 200, 1000, and 5000 as follows. If the sample has size *n* and the percentage of contamination is ε, then nε of the sample size is generated from model $N(\mu ,\sigma^{2})$ or $N(0,\sigma^{2})$ and the remaining n(1−ε) from a N(0,1) model. We use μ=1,5,10 and $\sigma^{2}=1$ in the $N(\mu ,\sigma^{2})$ model and $\sigma^{2}=4,9,16$ in the $N(0,\sigma^{2})$ model. The total variation distance was computed between the simulated data and the N(0,1) model. The Kullback-Leibler distance was calculated between the data generated from the aforementioned contamination models and a random sample of the same size *n* from N(0,1). When computing the mixture index of fit, we specified the component distribution as a normal distribution with initial mean 0 and variance 1. All simulations were carried out on a laptop computer with an Intel Core i7 processor and 64 bit Windows 7 operation system. The R packages used are presented in Table 1.

Table 2 and Table 3 present means and standard deviations of the total variation and Kullback-Leibler distances as a function of the contamination model and the sample size. To compute the total variation distance we use the R function “TotalVarDist” of the R package “distrEx”. It smooths the empirical distribution of the provided data using a normal kernel and computes the distance between the smoothed empirical distribution and the provided continuous distribution (in our case this distribution is N(0,1)). We note here that the package “distrEx” provides an alternative option to compute the total variation which relies on discretizing the continuous distribution and then computes the distance between the discretized continuous distribution and the data. We think that smoothing the data to obtain an empirical estimator of the density and then calculating its distance from the continuous density is a more natural way to handle the difference in scale between the discrete data and the continuous model. Lindsay [1] and Markatou et al. [3] discuss this phenomenon and call it discretization robustness. The Kullback-Leibler distance was computed using the function “KLD.matrix” of the R package “bioDist”.

We observe from the results of Table 2 and Table 3 that the total variation distance for small percentages of contamination is small and generally smaller than the Kullback-Leibler distance for both asymmetric and symmetric contamination models with a considerably smaller standard deviation. The above behavior of the total variation distance in comparison to the Kullback-Leibler manifests itself across all sample sizes used.

Table 4 presents the mixture index of fit computed using the R function “pistar.uv” from the R package “pistar” (https://rdrr.io/github/jmedzihorsky/pistar/man/; accessed on 5 June 2018). Since the fundamental assumption in the definition of the mixture index of fit is that the population on which the index is applied is heterogeneous and expressed via the two-point model, we only used the asymmetric contamination model for various values of the contamination distribution.

We observe that the mixture index of fit generally estimates well the mixing proportion ε. We observe (see Table 4) that when the second population is N(1,1) the bias associated with estimating the mixing (or contamination) population can be as high as 30.6%. This is expected because the population N(1,1) is very close to N(0,1) creating essentially a unimodal sample. As the means of the two normal components get more separated, the mixture index of fit provides better estimates of the mixing quantity and the percentage of observations that need to be removed so that N(0,1) provides a good fit to the remaining data points.

## 7. Discussion and Conclusions

Divergence measures are widely used in scientific work, and popular examples of these measures include the Kullback-Leibler divergence, Bregman Divergence [30], the power divergence family of Cressie and Read [31], the density power divergence family [32] and many others. Two relatively recent books that discuss various families of divergences are Pardo [33] and Basu et al. [34].

In this paper we discuss specific divergences that do not belong to the family of quadratic divergences, and examine their role in assessing model adequacy. The total variation distance might be preferable as it seems closest to a robust measure, in that if the two probability measures differ only on a set of small probability, such as a few outliers, then the distance must be small. This was clearly exemplified in Table 2 and Table 3 of Section 6. Outliers influence chi-squared measures more. For example, the Pearson’s chi-squared distance can be made dramatically larger by increasing the amount of data in a cell with small model probability $m_{\theta }\left(t\right)$. In fact, if there is data in a cell with model probability zero, the distance is infinite. Note that if data occur in a cell with probability, under the model, equal to zero, then it is possible that the model is not true. Still, even in this case, we might wish to use it on the premise that $m_{\theta }$ provides a good approximation.

There is a pressing need for the further development of well-tested software for computing the mixture index of fit. This measure is intuitive and has found many applications in the social sciences. Reiczigel et al. [35] discuss bias-corrected point estimates of π*, as well as a bootstrap test and new confidence limits, in the context of contingency tables. Well-developed and tested software will further popularize the dissemination and use of this method.

The mixture index of fit ideas were extended in the context of testing general model adequacy problems by Liu and Lindsay [9]. Recent work by Ghosh and Basu [36] presents a systematic procedure of generating new divergences. Ghosh and Basu [36], building upon the work of Liu and Lindsay [9], generate new divergences through suitable model adequacy tests using existing divergences. Additionally, Dimova et al. [37] use the quadratic divergences introduced in Lindsay et al. [2] and construct a model selection criterion from which we can obtain AIC and BIC as special cases.

In this paper, we discuss non-quadratic distances that are used in many scientific fields where the problem of assessing the fitted models is of importance. In particular, our interest centered around the properties and potential interpretations of these distances, as we think this offers insight into their performance as measures of model misspecification. One important aspect for the dissemination and use of these distances is the existence of well-tested software that facilitates computation. This is an area where further development is required.

## Figures and Tables

**Table 1:** Computer packages for calculating total variation, mixture index of fit, and Kullback- Leibler distances.

Information	Total Variation	Kullback-Leibler	Mixture Index of Fit
R package	distrEx	bioDist	pistar
R function	TotalVarDist	KLD.matrix	pistar.uv
Dimension	Univariate	Univariate	Univariate
Website	https://cran.r-project.org/web/packages/distrEx/	http://bioconductor.org/packages/release/bioc/html/bioDist.html	https://rdrr.io/github/jmedzihorsky/pistar/man/

**Table 2:** Means and standard deviations (SD) of the total variation (TV) and Kullback-Leibler (KLD) distances. Data are generated from the model (1−ε)N(0,1)+εN(μ,1) with μ=1,5,10. The sample size *n* is 200, 1000, 5000. The number of Monte Carlo replications is 500.

Contaminating Model	Percentage of Contamination (ε)	Summary	n=200		n=1000		n=5000	
			TV	KLD	TV	KLD	TV	KLD
*N*(1, 1)	0.01	Mean	0.144	0.224	0.065	0.048	0.029	0.008
		SD	0.017	0.244	0.007	0.051	0.004	0.009
	0.05	Mean	0.146	0.255	0.069	0.065	0.034	0.017
		SD	0.017	0.267	0.009	0.059	0.004	0.015
	0.1	Mean	0.149	0.323	0.076	0.088	0.047	0.026
		SD	0.017	0.343	0.009	0.073	0.005	0.018
	0.2	Mean	0.162	0.482	0.097	0.147	0.081	0.059
		SD	0.020	0.462	0.011	0.123	0.006	0.030
	0.3	Mean	0.181	0.616	0.128	0.215	0.117	0.102
		SD	0.022	0.528	0.013	0.150	0.007	0.044
	0.4	Mean	0.201	0.733	0.162	0.293	0.155	0.153
		SD	0.024	0.616	0.014	0.176	0.007	0.058
	0.5	Mean	0.232	0.937	0.198	0.392	0.192	0.207
		SD	0.026	0.735	0.014	0.203	0.007	0.067
*N*(5, 1)	0.01	Mean	0.149	0.577	0.070	0.338	0.034	0.231
		SD	0.017	0.373	0.008	0.131	0.004	0.063
	0.05	Mean	0.167	1.416	0.092	1.041	0.060	0.838
		SD	0.020	0.499	0.009	0.248	0.004	0.138
	0.1	Mean	0.196	2.392	0.126	2.002	0.103	1.731
		SD	0.020	0.609	0.010	0.335	0.004	0.219
	0.2	Mean	0.259	4.841	0.210	4.404	0.199	3.947
		SD	0.023	0.941	0.012	0.512	0.006	0.383
	0.3	Mean	0.336	7.924	0.302	7.305	0.297	6.652
		SD	0.028	1.182	0.014	0.730	0.007	0.569
	0.4	Mean	0.419	11.317	0.398	10.655	0.396	9.843
		SD	0.031	1.388	0.016	0.863	0.006	0.792
	0.5	Mean	0.506	15.045	0.495	14.443	0.494	13.573
		SD	0.035	1.768	0.016	1.027	0.007	0.999
*N*(10, 1)	0.01	Mean	0.149	0.352	0.070	0.129	0.034	0.082
		SD	0.017	0.275	0.008	0.071	0.004	0.024
	0.05	Mean	0.169	0.862	0.094	0.713	0.061	0.705
		SD	0.018	0.408	0.009	0.178	0.004	0.093
	0.1	Mean	0.197	1.898	0.128	1.850	0.105	1.854
		SD	0.020	0.593	0.010	0.261	0.004	0.132
	0.2	Mean	0.259	4.685	0.211	4.640	0.202	4.638
		SD	0.026	0.968	0.013	0.423	0.006	0.253
	0.3	Mean	0.340	8.393	0.305	8.055	0.300	7.909
		SD	0.029	1.391	0.014	0.631	0.007	0.388
	0.4	Mean	0.420	12.209	0.402	11.846	0.401	11.653
		SD	0.031	1.433	0.014	0.657	0.007	0.448
	0.5	Mean	0.515	16.544	0.503	16.041	0.501	15.841
		SD	0.032	1.499	0.016	0.730	0.007	0.432

**Table 3:** Means and standard deviations (SD) of the total variation (TV) and Kullback-Leibler (KLD) distances. Data are generated from the model $\left(1-\varepsilon \right)N\left(0,1\right)+\varepsilon N\left(0,\sigma^{2}\right)$ with $\sigma^{2}=4,9,16$. The sample size *n* is 200, 1000, 5000. The number of Monte Carlo replications is 500.

Contaminating Model	Percentage of Contamination (ε)	Summary	n=200		n=1000		n=5000	
			TV	KLD	TV	KLD	TV	KLD
*N*(0, 4)	0.01	Mean	0.145	0.263	0.066	0.068	0.030	0.021
		SD	0.017	0.250	0.008	0.058	0.003	0.014
	0.05	Mean	0.147	0.497	0.069	0.204	0.034	0.079
		SD	0.017	0.391	0.008	0.130	0.004	0.036
	0.1	Mean	0.154	0.778	0.076	0.368	0.044	0.181
		SD	0.018	0.527	0.008	0.168	0.004	0.062
	0.2	Mean	0.166	1.275	0.094	0.712	0.071	0.426
		SD	0.020	0.639	0.010	0.255	0.005	0.108
	0.3	Mean	0.182	1.797	0.118	1.067	0.101	0.671
		SD	0.021	0.738	0.012	0.324	0.006	0.158
	0.4	Mean	0.201	2.320	0.144	1.407	0.133	0.924
		SD	0.021	0.875	0.012	0.403	0.006	0.198
	0.5	Mean	0.220	2.766	0.173	1.755	0.164	1.164
		SD	0.025	0.932	0.013	0.450	0.006	0.219
*N*(0, 9)	0.01	Mean	0.146	0.369	0.067	0.122	0.031	0.046
		SD	0.018	0.348	0.007	0.089	0.003	0.022
	0.05	Mean	0.154	0.839	0.074	0.490	0.040	0.321
		SD	0.017	0.477	0.008	0.187	0.004	0.081
	0.1	Mean	0.164	1.414	0.087	0.945	0.058	0.661
		SD	0.018	0.602	0.009	0.256	0.005	0.120
	0.2	Mean	0.189	2.529	0.120	1.748	0.101	1.300
		SD	0.021	0.801	0.011	0.366	0.005	0.188
	0.3	Mean	0.216	3.529	0.161	2.526	0.149	1.954
		SD	0.023	0.957	0.012	0.466	0.006	0.276
	0.4	Mean	0.252	4.608	0.205	3.444	0.196	2.660
		SD	0.026	1.071	0.014	0.549	0.006	0.339
	0.5	Mean	0.286	5.630	0.250	4.289	0.244	3.423
		SD	0.026	1.123	0.014	0.657	0.007	0.406
*N*(0, 16)	0.01	Mean	0.146	0.429	0.067	0.166	0.031	0.078
		SD	0.016	0.374	0.007	0.100	0.003	0.032
	0.05	Mean	0.156	1.073	0.078	0.716	0.044	0.511
		SD	0.017	0.514	0.008	0.203	0.004	0.088
	0.1	Mean	0.169	1.774	0.094	1.281	0.066	0.981
		SD	0.019	0.606	0.008	0.277	0.005	0.142
	0.2	Mean	0.200	3.160	0.137	2.383	0.120	1.927
		SD	0.021	0.800	0.011	0.408	0.005	0.218
	0.3	Mean	0.239	4.471	0.187	3.532	0.177	2.937
		SD	0.025	1.045	0.013	0.485	0.006	0.278
	0.4	Mean	0.280	5.812	0.242	4.822	0.235	4.044
		SD	0.026	1.125	0.014	0.589	0.007	0.355
	0.5	Mean	0.331	7.537	0.298	6.145	0.293	5.218
		SD	0.029	1.274	0.015	0.693	0.007	0.433

**Table 4:** Means and standard deviations (SD) for the mixture index of fit. Data are generated from an asymmetric contamination model of the form (1−ε)N(0,1)+εN(μ,1), μ=1,5,10 with sample sizes, *n*, of 1000, 5000. The number of Monte Carlo replications is 500.

Percentage of Contamination ε	Summary	N(1,1)		N(5,1)		N(10,1)	
		n=1000	n=5000	n=1000	n=5000	n=1000	n=5000
0.1	Mean	0.180	0.160	0.223	0.213	0.837	0.934
	SD	0.045	0.044	0.041	0.040	0.279	0.198
0.2	Mean	0.184	0.172	0.288	0.287	0.433	0.521
	SD	0.044	0.042	0.036	0.036	0.144	0.240
0.3	Mean	0.189	0.179	0.344	0.346	0.314	0.317
	SD	0.047	0.039	0.028	0.024	0.016	0.012
0.4	Mean	0.194	0.186	0.436	0.436	0.410	0.413
	SD	0.044	0.034	0.026	0.021	0.017	0.011
0.5	Mean	0.194	0.185	0.529	0.533	0.511	0.512
	SD	0.047	0.035	0.024	0.020	0.017	0.010
